# A Machine Learning Approach to Predict Gene Regulatory Networks in Seed Development in Arabidopsis

**DOI:** 10.3389/fpls.2016.01936

**Published:** 2016-12-23

**Authors:** Ying Ni, Delasa Aghamirzaie, Haitham Elmarakeby, Eva Collakova, Song Li, Ruth Grene, Lenwood S. Heath

**Affiliations:** ^1^Department of Computer Science, Virginia Polytechnic Institute and State UniversityBlacksburg, VA, USA; ^2^Genetics, Bioinformatics and Computational Biology, Virginia Polytechnic Institute and State UniversityBlacksburg, VA, USA; ^3^Department of Plant Pathology, Physiology, and Weed Science, Virginia Polytechnic Institute and State UniversityBlacksburg, VA, USA; ^4^Department of Crop and Soil Environmental Sciences, Virginia Polytechnic Institute and State UniversityBlacksburg, VA, USA

**Keywords:** gene regulatory network, Arabidopsis, gene expression, support vector machines, cluster, binding site

## Abstract

Gene regulatory networks (GRNs) provide a representation of relationships between regulators and their target genes. Several methods for GRN inference, both unsupervised and supervised, have been developed to date. Because regulatory relationships consistently reprogram in diverse tissues or under different conditions, GRNs inferred without specific biological contexts are of limited applicability. In this report, a machine learning approach is presented to predict GRNs specific to developing *Arabidopsis thaliana* embryos. We developed the Beacon GRN inference tool to predict GRNs occurring during seed development in Arabidopsis based on a support vector machine (SVM) model. We developed both global and local inference models and compared their performance, demonstrating that local models are generally superior for our application. Using both the expression levels of the genes expressed in developing embryos and prior known regulatory relationships, GRNs were predicted for specific embryonic developmental stages. The targets that are strongly positively correlated with their regulators are mostly expressed at the beginning of seed development. Potential direct targets were identified based on a match between the promoter regions of these inferred targets and the *cis* elements recognized by specific regulators. Our analysis also provides evidence for previously unknown inhibitory effects of three positive regulators of gene expression. The Beacon GRN inference tool provides a valuable model system for context-specific GRN inference and is freely available at https://github.com/BeaconProjectAtVirginiaTech/beacon_network_inference.git.

## Introduction

Elucidating the topology of gene regulatory networks (GRNs) is fundamental to understanding how transcription factors (TFs) regulate gene expression and the complexity of interdependencies among genes. Potential TF target relationships can be identified by using chromatin immunoprecipitation with DNA microarray (ChIP-chip; Junker et al., 2010), ChIP-sequencing (Park, 2009), or protein-binding microarrays (Berger and Bulyk, 2009). However, these wet-lab experiments are technically challenging, financially demanding, and time consuming (Penfold and Wild, 2011). Many computational approaches have been proposed to infer GRNs using gene expression levels. With the advent of high-throughput transcriptome methods such as RNA sequencing (RNA-seq), computational inference of a regulatory network on a genome scale has been made more feasible. Inference through computational methods is convenient, and there are various ways to validate the results (Schrynemackers et al., 2014; Patel and Wang, 2015).

GRNs can be depicted as directed graphs, where TFs and genes are nodes and interactions or regulations are edges. Early computational work used unsupervised approaches, such as weighted gene correlation network analysis (WGCNA) (Langfelder and Horvath, 2008), the context likelihood of relatedness algorithm (CLR; Faith et al., 2007), or trustful inference of gene regulation using stability selection (TIGRESS; Haury et al., 2012). These methods predict networks exclusively from expression data, and they can be used when gene regulation information is limited. However, as large numbers of TF-target interactions become available, using these prior known interactions is likely to improve prediction accuracy. In one of the most recent and largest comparisons of GRN inference methods (Maetschke et al., 2014), 17 unsupervised methods were compared with a supervised method—the support vector machine (SVM)—in three different experimental conditions using both simulated and experimental data sets. It was found that the supervised method performed the best, except for knockout experiments, when it was surpassed by the *Z*-score method. Similar results have been published (Mordelet and Vert, 2008) where the supervised inference of regulatory networks (SIRENE) method was compared with four unsupervised methods, CLR, the algorithm for the reconstruction of accurate cellular networks (ARACNE), relevance networks (RN), and a Bayesian network, using an *Escherichia coli* benchmark data set (Faith et al., 2007). It was concluded that the supervised method significantly outperformed unsupervised methods. Recently, Gillani et al. (2014) compared the performance of four kernel functions based on SVM with CLR on simulated *E. coli* microarray data sets. They concluded that SVM with the Gaussian kernel inferred small networks (<200 nodes) with the highest prediction accuracy, while CLR outperformed all other methods for inferring networks with an increased number of nodes (about 500).

These methods are referred to as non-targeted (Aoki et al., 2007) or condition independent because they provide an overall network structure, using data obtained across many conditions and are not specific to a biological process of interest. The major drawback of these methods is that gene interactions occurring under specific conditions or during a particular biological process are easily missed, which, however, can be alleviated by using data that are relevant to a specific biological condition (Serin et al., 2016). Here, we focus on the GRNs related to the model plant *Arabidopsis thaliana* during embryo development.

Seed and embryo development are important and interconnected complex processes in the life cycle of flowering plants and can be divided into three major stages (Meinke, 1995; Baud et al., 2008; Lafon-Placette and Kohler, 2014). The first stage is embryogenesis, when the basic body of a plant is established. The second stage is maturation, when seed storage compounds are synthesized and accumulate in the embryo and different parts of a seed. The third stage is the acquisition of desiccation tolerance followed by dormancy. Seed development is tightly regulated by plant growth regulators, light, temperature, and stress (Nakashima and Yamaguchi-Shinozaki, 2013; Sreenivasulu and Wobus, 2013; Verma et al., 2016). In Arabidopsis, genetic studies have identified several key regulators that regulate distinct aspects of seed development (Jia et al., 2014). The LEC1/AFL (LAFL) TF network is composed of TFs including B3 domain TFs ABSCISIC ACID (ABA)-INSENSITIVE3 (ABI3), FUSCA3 (FUS3), and LEAFY COTYLEDON2 (LEC2, AFL), and two LEC1-type HAP3 family CCAAT-binding factors, LEC1 and LEC1-LIKE (Jia et al., 2013). These LAFL TFs, together with many overlapping and unique downstream targets, constitute a complex transcriptional regulatory network that regulate seed development (Mendes et al., 2013). To date, these LAFL TFs have been primarily associated with the activation of their respective target genes (Jia et al., 2014). Previous efforts to infer GRNs operating in Arabidopsis seeds, such as the seed-specific network associated with dormancy and germination established by Bassel et al. (2011) that used the WGCNA algorithm and 138 samples from mature imbibed Arabidopsis seeds, constitute progress toward understanding gene interactions in seeds. However, interactions of downstream targets of the well-known core LAFL TFs and related TFs are only partially understood in seed development. Here, we propose to use tissue-specific SVMs to investigate regulation during seed development using the expression data of genes expressed at particular developmental stages.

For the inference algorithm, we developed the Beacon inference tool using supervised SVM. In the context of supervised methods, global and local approaches are two main categories that have been reported in the literature to transform the network inference problem to a classification problem (Vert, 2010). Global approaches consider each pair of genes as a single object, and the classification is performed on these objects (Ben-Hur and Noble, 2005; Maetschke et al., 2014). Therefore, a feature vector has to be constructed for each gene pair. Instead of focusing on gene pairs, local approaches divide the inference problem into several smaller classification problems. Each small classification problem corresponds to a TF of interest, aiming to infer all target genes that are associated with this TF (Mordelet and Vert, 2008; Gillani et al., 2014). The resulting networks for all TFs are combined to form the complete network. We estimated a global model for all gene pairs and local models for each TF and its target genes in the embryo development data set. We evaluated the prediction accuracy of the SVM using two widely used kernel functions in comparison to an unsupervised method (CLR). Being a supervised method, SVM requires a list of known regulatory relationships between TFs and targets to train a classifier, which is then used to predict unknown connections. For the TFs, we considered ABI3, FUS3, LEC2, and LEC1, as they represent an integral part of the LAFL regulatory network (Jia et al., 2013). Some previous studies have been dedicated to developing suitable and accurate approaches for predictions, but most of them lack adequate investigation and explanation of the prediction results (Mordelet and Vert, 2008; Gillani et al., 2014). Thus, analyzing the inferred network is another key part of our work. After clustering the target expression profiles to analyze co-expressed genes, promoter regions of the targets were scanned to search for the respective *cis* elements of the relevant TFs. Further investigation of the functional categories that were enriched in each cluster revealed meaningful insights into the regulation of Arabidopsis embryo development.

In summary, first, the supervised and unsupervised methods are described in Section Materials and Methods, before evaluating their prediction accuracies on Arabidopsis seed development gene expression data (Sections Algorithm Evaluation and Comparison and Network Prediction). We choose to compare the supervised SVM method and the un-supervised CLR method because it has been demonstrated that, in large networks, CLR but not other supervised methods can out-perform SVM (Gillani et al., 2014). Second, clustering (Section Statistical Analysis), binding site identification, comparison with other experimental data, and data mining of the prediction results (Section Comparison of Target Genes Predicted by the Beacon GRN Inference Tool with Those Identified in GeneMania for ABI3, LEC1, and FUS3) are presented. The LAFL TFs are known primarily as positive regulators of gene expression (Jia et al., 2013). The data mining yielded unexpected evidence that ABI3 may have negative regulatory influence on specific groups of genes that are expressed during late seed filling stages of embryo development (Section Discussion).

## Materials and methods

### Data preparation

RNA-Seq-based transcriptomics data related to differentially expressed genes in *A. thaliana* (Col-0) embryo development were used. This data set contains the expression profiles of a total of 53,989 transcripts expressed in embryos of different ages represented by seven time points (7, 8, 10, 12, 13, 15, and 17 days after pollination (DAP) in three biological and four technical replicates; Schneider et al., 2016). Expression of these transcripts was normalized using fragments per kilobase of transcript per million mapped reads (FPKM). The gene expression levels in FPKM was calculated by summing the FPKM expression values from all splice variants (transcripts originating from the same gene) for a given gene for each time point. Limma analysis (Ritchie and Nesmith, 1991; Smyth, 2005; Ritchie et al., 2015) was then applied to identify the genes that are differentially expressed at least at one time point with respect to its previous time point (Section Limma Analysis) as described (Schneider et al., 2016). We found that 7376 genes were significantly differentially expressed at least one time point out of a total of 32,836 Arabidopsis genes represented in the data set. Regulons for each LAFL TF were obtained by compiling experimentally confirmed regulatory relationships between four LAFL regulators and their target genes. Specifically, the regulation data sets for LEC1, LEC2, FUS3, and ABI3 were extracted from Braybrook et al. (2006), Junker et al. (2010), Mönke et al. (2012), Wang and Perry (2013). Information concerning experimental design and the number of target genes are summarized in Table 1. As only 14 target genes were reported to be regulated by LEC2, no statistically significant results can be inferred from such a small number of relationships, so the data set for LEC2 was not used in our study.

**Table 1:** **Source of positive examples in prior knowledge**.

**Data sets**	**Number of samples**	**Number of targets**	**Tissues**	**Number of differentially expressed targets**	**References**
LEC1^*^	16	356	Two-week old seedlings	174	Junker et al., 2010
LEC2^**^	8	14	Eight-day old seedlings	14	Braybrook et al., 2006
FUS3^*^	1	1218	Embryonic culture expressing FUS3	508	Wang and Perry, 2013
ABI3^*^	40	98	Two-week old seedlings	94	Mönke et al., 2012

*Number of target genes of LEC1, LEC2, FUS3, and ABI3, number of samples, techniques and tissues extracted from literature are listed, and the number of differentially expressed targets is identified*.

* ChIP-chip and

** *Microarray experiments*.

### Methods

#### Limma analysis

Instead of using FPKM values, Limma requires raw counts as input data, and the raw counts are the number of reads overlapping a given gene. In the Limma pipeline, the VOOM package (Law et al., 2014) was first used to normalize the counts. Empirical Bayes, moderated *t*-statistics, and their associated *p*-values were then used to assess the significance of the observed expression changes between two consecutive time points. Genes with adjusted *p* < 0.05 were declared to be differentially expressed.

#### Performance of inference algorithms

To evaluate the performance of inference algorithms, receiver operator characteristic (ROC) curves and the computed area under the receiver operator characteristic curve (AUC) were used as described (Mordelet and Vert, 2008; Haynes and Brent, 2009; Kiani and Kaderali, 2014; Omranian et al., 2016). ROC curves show the true positive rates over the full range of false positive rates at different thresholds, and AUC quantifies the quality of the classifier. The AUC value represents the probability based on the fact that the classifier ranks a randomly chosen positive instance higher than a randomly chosen negative instance. AUC is a portion of a unit square and hence its value will always be between 0 and 1. An AUC above 0.5 is expected for a realistic classifier as it should perform better than random guessing, while an AUC of 1 indicates perfect performance (Fawcett, 2006). An unsupervised method does not require any parameter optimization. For supervised methods, on the other hand, cross validation (Devijver and Kittler, 1982) is usually applied and parameters are optimized on the training data only (Section Support Vector Machines).

#### Support vector machines

A variety of different supervised machine learning approaches are available. SVM was chosen here as it has been demonstrated to outperform the other methods of GRN inference in some significant circumstances (Mordelet and Vert, 2008; Maetschke et al., 2014). We used the Python implementation of an SVM, sklearn.svm, published by Pedregosa et al. (2011). Here, we compared the performance of global and local SVMs. Let *t* be the target gene, *r* be the regulator, *i* = *1*, …, *k* be the time point, and *e*(*t*_*i*_) and *e*(*r*_*i*_) be the expression levels of genes *t* and *r* at time point *i*, respectively; feature vector of the gene pair (*r, t*) is defined as **x**. The first way of constructing **x** is to directly concatenate the expression data of regulator and target: **x** = (*e*(*r*_1_), …, *e*(*r*_*k*_), *e*(*t*_1_), …, *e*(*t*_*k*_))^*T*^. This belongs to the global approach because each gene pair is treated as a single object and only one SVM is used for training predictions. The second way is $\text{x}\;=\;{\left(\log\frac{e\left(t_{2}\right)}{e\left(t_{1}\right)},\;\ldots ,\;\log\frac{t_{k}}{e\left(t_{k-1}\right)}\right)}^{T}$, which belongs to the local approach because each regulator is treated as a separate SVM.

The kernel function is a fundamental component of an SVM algorithm. Given *r* as the regulator and *n* target genes *t*_1_, …, *t*_*n*_, the gene pairs (*r, t*_1_), (*r, t*_2_), …, (*r, t*_*n*_) belong to two classes +1 and −1. Class +1 means that gene *r* regulates gene *t*, while class −1 means that gene *r* does not regulate gene *t*. The optimization algorithm of SVM will construct a hyperplane that separates these two classes, and the optimal hyperplane maximizes the distance of the closest point to the hyperplane. We applied the SVC method for soft-margin SVMs implemented in the scikit-learn package (Pedregosa et al., 2011). In general, soft-margin SVM solves a constrained optimization problem which allows misclassification by introducing a slack variable s_*i*_ for each training variable. The objective function and constraints is in the following form (Ben-Hur et al., 2008):

$$\begin{array}{l} \underset{w,b,s}{\text{minimize}}\frac{1}{2}||w|{|}^{2}+C\overset{n}{\underset{i=1}{\sum }}s_{i}\\ \text{subject to}:y_{i}\left(w^{\text{T}}x_{i}+b\right)\geq 1-s_{i},\\ s_{i}\geq 0,\text{ for i}=1,\ldots \text{n}. \end{array}$$

In these formulas, **x**_*i*_ denotes the feature vector of the gene pair (*r, t*_*i*_), *w* is the weight vector, and *b* is the bias parameter. Here, *y*_*i*_ is the label of training data, with *y*_*i*_ = 1 for positive training samples and *y*_*i*_ = −1 for negative training samples. Note that s_*i*_ is the slack variable. For those data points that fall on the correct side of the decision boundary, s_*i*_ ≤ 1, whereas when data points fall on the wrong side of the decision boundary, s_*i*_ > 1. The parameter C can be viewed as a relative weight of the slack variables and the *w* vector (Bishop, 2007).

To classify new data points, a scoring function is evaluated. For example, let **x**′_*j*_ denote the feature vector of a new gene pair (*r, t*_*j*_), the kernel function between **x**_*i*_ and **x**′_*j*_ is *k*(**x**_*i*_, **x**′_*j*_). An SVM estimates a scoring function for any new gene pair (*r, t*_*j*_) in the following form:

$$\begin{array}{l} f\left(x\prime_{j}\right)=\sum_{i=1}^{n}y_{i}\alpha_{i}k\text{​}\left(x_{i},x\prime_{j}\right)+b \end{array}$$

The α_*i*_ in the equation are Lagrange multipliers, which are selected by the SVM algorithm to obtain large positive scores for genes in the +1 class and large negative scores for genes in the −1 class in the training set. After α_*i*_ is obtained, the scoring function $f\left(x\prime_{j}\right)$ can then be used to classify genes from unknown classes in the test set. To find the SVM kernel with the best performance, experiments were conducted to evaluate the following linear and Gaussian kernel functions. Though there are many kernel functions available, these two functions are mostly used in gene network inference and have proved to perform well in previous studies (Mordelet and Vert, 2008; Cerulo et al., 2010; Maetschke et al., 2014).

1. Linear Kernel
  The linear kernel is the simplest kernel function for an SVM. The linear kernel is defined as the dot product of two vectors **x** and $x\prime_{j}$ with addition of a constant *c*:
  $$\begin{array}{l} k\left(\text{x},\;{\text{x}}^{\prime }\right)={\text{x}}^{T}{\text{x}}^{\prime }+c. \end{array}$$
2. Gaussian Kernel
  The Gaussian kernel is a radial basis kernel function or RBF kernel defined by:
  $$\begin{array}{l} k\left(\text{x},\;\text{x}\prime \right)=exp\left(-\gamma ||\text{x}-\text{x}\prime |{|}^{2}\right), \end{array}$$
  where $\gamma =\frac{1}{2\sigma^{2}}$ and σ > 0. Here, σ is a parameter that controls the width of the Gaussian kernel. If σ is underestimated, the kernel becomes more local and forms a greater curvature of the decision surface, which makes the radius of the area of influence of the support vectors too small so that it only includes the support vector itself. If overestimated, the model behaves similarly to the linear model, resulting in a failure to capture the shape of the data.
  With a very high value of C, the training mistakes have very high cost. Here, we chose *C* = 1000 to train all SVMs. This choice was also used by SIRENE (Mordelet and Vert, 2008). The choice of $\gamma =\frac{1}{number\;of\;samples}$ was used according to the default settings by sklearn.svm (Pedregosa et al., 2011).

As a supervised learning method, SVM needs both positive and negative examples in a training set. Positive examples are known relationships between well-studied regulators and their targets as described in Section Limma Analysis. However, the known regulatory relationship data sets contain the genes that are not differentially expressed. Because we aim to predict regulatory relationships among the differentially expressed genes, the evaluation should also be done on this set. Therefore, we divided the positive examples into two subsets, differentially expressed and not differentially expressed positive examples, according to whether the target gene is differentially expressed (Table 1). For negative examples, there is little information about a regulator not regulating expression of specific genes. In this paper, we randomly chose a subset of regulator-target gene pairs that were absent from the prior known regulatory relationship data sets as the negative example set. This is based on the premise that transcription of the majority of expressed genes that were not identified as part of the corresponding regulons is likely not regulated by a given TF. This subset contains the same number of genes as in the positive example set. A three-fold cross validation was done by randomly splitting the differentially expressed positive and negative example sets into three subsets, training on two of the subsets plus the stably expressed positive examples, and evaluating the prediction on the last subset. This process was repeated three times, testing successively on each subset. The prediction quality was averaged over all three iterations.

#### CLR

The performance of SVM was compared with that of the CLR method (Faith et al., 2007). CLR is a widely used unsupervised learning method for gene network inference. The CLR method was implemented according to Faith et al. (2007) using the default parameters. CLR extends the relevance network method (Butte and Kohane, 2000) and makes use of mutual information (MI) values. MI between two discrete random variables *X*_*i*_ and *X*_*j*_ is defined as

$$\begin{array}{l} I\left(X_{i},\;X_{j}\right)=\sum_{x_{j}\in X_{j}}\sum_{x_{j}\in X_{j}}p\left(x_{i},x_{j}\right)\log\frac{p\left(x_{i},x_{j}\right)}{p\left(x_{i}\right)p\left(x_{j}\right)}, \end{array}$$

where *p*(*x*_*i*_) and *p*(*x*_*j*_) are marginal probabilities, and *p*(*x*_*i*_, *x*_*j*_) is the joint probability distribution of *X*_*i*_ and *X*_*j*_.

CLR calculates the MI values between all gene pairs and produces a MI matrix **M**, where **M**_*ij*_ is the MI value between gene *i* and gene *j*. The background MI distribution is then taken into account to estimate the interaction between genes *i* and *j*. The background distribution consists of two sets of MI values: all MI values for gene *i*, **M**_*ik*_, *k* = 1, …, *n*, and all MI values for gene *j*, **M**_*jk*_, *k* = 1, …, *n*. In the CLR technique, it is assumed that the interactions with MI that deviate most from the background distribution are the most probable interactions. Thus, a maximum *z*-score is computed for each gene *i* as

$$\begin{array}{l} z_{i}=\underset{j}{\max}\left(0,\frac{{\text{M}}_{ij}-\mu }{\sigma_{i}}\right), \end{array}$$

where μ and σ are the mean value and standard deviation, respectively, of the MI values **M**_*ik*_. The final form of the CLR likelihood estimation is

$$\begin{array}{l} w_{i,j}=\sqrt{z_{i}^{2}+z_{j}^{2}}. \end{array}$$

Putative regulator-gene interactions are then ranked by decreasing *w*_*i, j*_.

In the spirit of the DREAM Challenge (Marbach et al., 2012), we did additional analysis to compare our model to other supervised predictive models. We compared our model, which is based on RBF-SVM, with nine supervised models in terms of area under curve AUC. The results showed that our model is ranked first for the ABI3 and LEC1 data sets and comes just barely second in the FUS3 data set. See Supplementary Images 1–6 for results.

We believe that, given the small data sets that we have, many models can achieve comparable results. SVM is known for good generalization, ease of incorporating non-linearity through changing the kernels, a small number of hyper-parameters, and achieving state of the art performance in many contexts. This makes SVM a good choice for fitting our data.

#### Clustering

To analyze target genes and visualize their expression patterns, we grouped these genes by similar expression profiles using the *k*-means clustering algorithm (MacQueen, 1967), as implemented in Python (Pedregosa et al., 2011). It is a partition-based clustering method that can automatically partition a data set into *k* groups. Given a predetermined number *k*, and a set of gene expression values **x**_*1*_, **x**_*2*_, …, **x**_*n*_, where each gene expression value is a *k*-dimensional vector, the goal is to minimize the objective function

$$\begin{array}{l} E=\sum_{i=1}^{k}\sum_{x\in S_{i}}{\left|x-\mu_{i}\right|}^{2}, \end{array}$$

where μ_*i*_ is the centroid of cluster *S*_*i*_. Thus, *E* is to minimize the sum of squared distances (Euclidean distance) of gene expression values from their cluster centers. It proceeds by randomly choosing *k* cluster centers and then iteratively updating them as follows:

1. Each gene is assigned to its closest cluster center.
2. Each cluster center is updated to the mean of its constituent genes.

The algorithm converges when there is no further change in assignment of genes to clusters.

#### Direct targets

First, the CIS-BP (Catalog of Inferred Sequence Binding Preferences) database, which is one of the motif databases available in the MEME Web site (Bailey et al., 2009; http://meme-suite.org/tools/meme), was searched for the binding sites for each regulator to identify putative direct targets of the LAFL regulators. Second, upstream sequences (3000 bp or up to the next gene) were identified for all inferred target genes at the TAIR Web site (TAIR 10) https://www.arabidopsis.org/ (Berardini et al., 2015). Third, the FIMO (find individual motif occurrences) algorithm (Grant et al., 2011) was used with *p*-value output threshold setting of 1 × 10^−4^ to identify promoter sequences containing the binding sites to classify such genes as direct targets. To infer a further set of regulatory relationships, the TAIR database, specifically, the direct targets were searched for TFs, referred to as secondary TFs. This direct target analysis was repeated to predict the direct targets of the secondary TFs among the indirect targets of the primary TFs.

#### Experimental procedure

**Figure 1:**
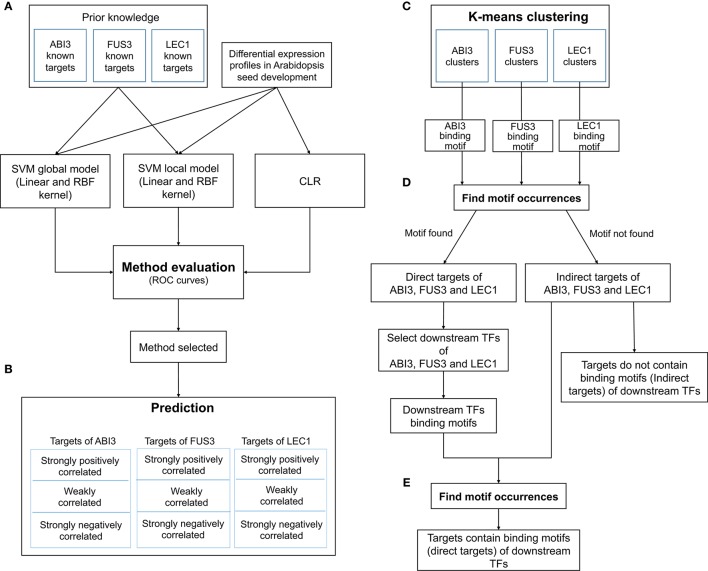
**Beacon GRN inference and validation workflow**. Five phases: method comparison **(A)**, prediction **(B)**, k-means clustering **(C)**, identify the targets contain binding motifs **(D)**, and identify targets contain the downstream TF binding motifs **(E)**. K-means clustering is done by combining known and predicted strongly correlated targets.

**Figure 2:**
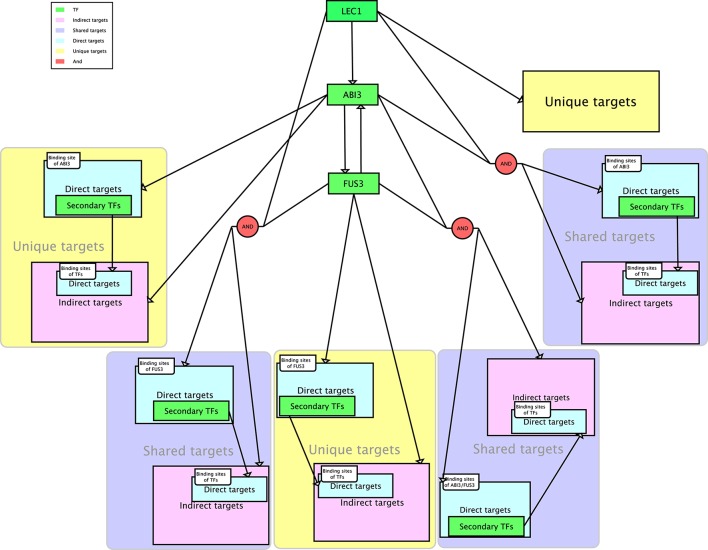
**The proposed network**. The diagram is drawn in Systems Biology Graphical Notation (SBGN) format (Le Novere et al., 2009). LEC1, FUS3, and ABI3 represent three master regulators, with ABI3 directly controlled by LEC1 and ABI3 and FUS3 mutually regulated.

The workflow for the GRN inference tool involved five phases, namely, comparison, prediction, clustering, searching for direct and indirect targets of regulators, and searching for direct and indirect targets of secondary TFs (Figure 1). The purpose of the comparison phase was to generate the ROC curve using the supervised method with global and local SVMs and the unsupervised method CLR (Figure 1A). To train the SVM classifiers, two types of inputs were required. The first input was a list of gene names and their expression levels for testing and training the classifiers. The second input was a list of positive and negative examples. An SVM classifier was trained for each regulator (ABI3, FUS3, and LEC1) based on the known target genes and non-target genes. For the global model, the three sub-problems were combined to obtain one problem, where a global SVM classifier was trained based on all known regulatory relationships. The list of testing regulatory relationships was assigned into different classes according to the trained SVM. This process was repeated for each kernel. Because the CLR algorithm does not require a training data set, the final ROC curve was generated on all genes simultaneously. The approach with the highest accuracy was used to predict new target genes of these regulators (Figure 1B). This analysis yielded three networks with ABI3, FUS3, and LEC1 as regulatory nodes. The target genes controlled by single or multiple regulators were identified. The following procedures are all related to individual networks. First, Pearson correlation was performed to determine correlation coefficients between the expression levels of the targets and their corresponding regulator. A threshold of 0.6 was chosen to retain strongly correlated targets and filter out targets with weakly correlated expression profiles. Second, the known and predicted strongly positively correlated target genes were grouped based on their expression patterns (Figure 1C). Third, the FIMO algorithm (Grant et al., 2011) was used to search for the direct targets in each cluster using relationships between co-expressed targets and their regulators (Figure 1D). Finally, the secondary TFs and their binding motifs were identified among the direct targets within each cluster, and FIMO was utilized again on indirect targets in each cluster to predict the direct targets of these secondary TFs (Figure 1E). As reviewed by Jia et al. (2014), LEC1 positively regulates ABI3, and ABI3 and FUS3 are mutually regulated. Combining these combinatorial relationships with our inferred three sub-networks yielded the entire network (Figure 2).

## Results

### Algorithm evaluation and comparison

**Figure 3:**
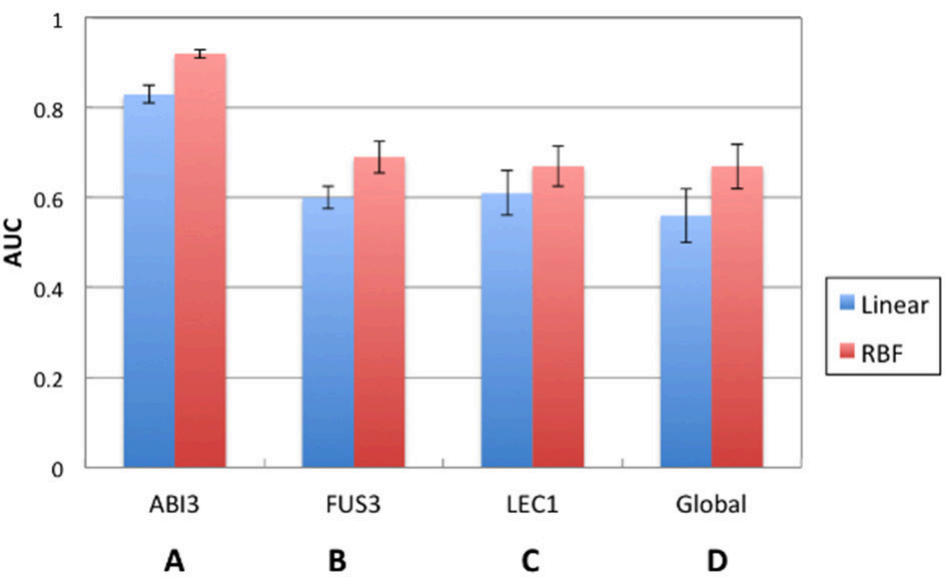
**Comparison of performance between SVM local models and global model**. **(A)** ABI3, **(B)** FUS3, and **(C)** LEC1 represent represent local models with each of them as a separate SVM. **(D)** Global model trains one SVM for all the TF-target pairs.

**Figure 4:**
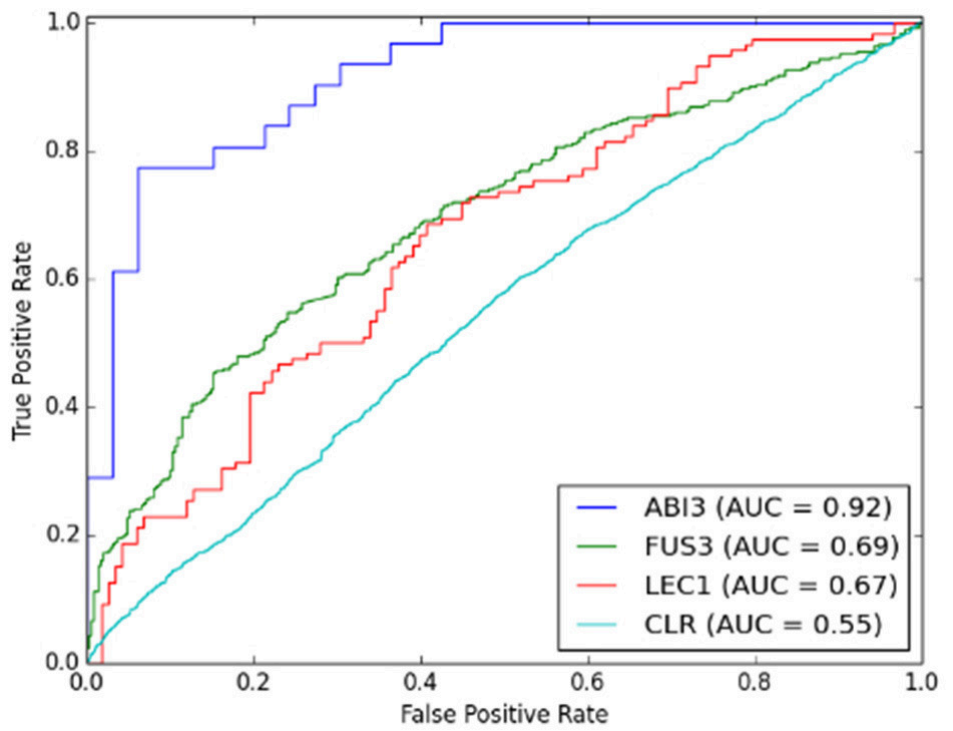
**Comparison of performance between SVM local models and CLR algorithm**.

SVM performance was evaluated prior to comparing CLR with the best performing SVM model. Figure 3 shows the comparison between the prediction accuracies measured by AUC for linear and RBF kernel SVMs. Figures 3A–C are the results of local models. Among all three regulators, the SVM of ABI3 with AUC approximately 0.9 performed the best. Figure 3D shows the result of the global model, which performed worse than the ABI3 model, but was comparable with FUS3 and LEC1. The performance of the two kernels was comparable as they had similar AUC values with the RBF kernel performing better than the linear kernel for all four cases. The reason for the poor performance of the global model is in its failure to capture the unique characteristics of different regulators that are well captured by the local models. Different regulators may have different modes of regulatory mechanism, and, as such, it is difficult to learn all different features in one SVM. Furthermore, as summarized in Table 1, FUS3 has 1045 known target genes, which exceeds the known targets of the other two selected TFs. Hence, the majority of the positive examples represent FUS3 regulatory relationships, while FUS3 regulatory relationships are minor in the negative example set. As a consequence, the SVM classifier may simply capture the features of FUS3 regulatory relationships as positives and considers all features different from these relationships as negatives. Because the local models appeared to be more meaningful and powerful than the global model, our focus was on the local model with the RBF kernel. The SVM local RBF model was then compared to the CLR algorithm, which, with the prediction accuracy 55%, performs much worse than the supervised model (Figure 4).

In summary, our evaluation of the methods indicates that a local SVM model with RBF kernel is the most suitable method for predicting regulatory networks related to the three regulators using gene differential expression in developing Arabidopsis embryos. We refer to this approach as the Beacon GRN inference tool.

### Network prediction

As described in Section Algorithm Evaluation and Comparison, ABI3, FUS3, and LEC1 models were treated as separate SVMs to predict networks based on all differentially expressed genes. The predicted networks were then combined to make one network.

The positive examples used in this analysis were the known targets listed in Table 1, genes which were expressed during seed development. We employed 98, 1045, and 353 positive examples and the same number of negative examples as the training sets for ABI3, FUS3, and LEC1, respectively. The Beacon GRN inference tool predicted 1064, 2569, and 3836 targets for ABI3, FUS3, and LEC1, respectively (Table 2). The targets regulated by unique and multiple regulators were then identified, including the overlaps.

**Table 2:** **Number of predicted and unique targets for each regulator**.

**Regulator**	**Number of predicted targets**	**Number of unique targets**
ABI3	1064	275
FUS3	2596	862
LEC1	3836	1732

### Statistical analysis

To further filter the results, targets whose expression levels were most strongly positively correlated with the expression levels of their related regulators were identified (Table 3). Approximately 50% of the FUS3 and LEC1 targets were discarded with the correlation coefficient threshold set at 0.6. The remaining, strongly positively correlated, targets were used for the following analysis.

**Table 3:** **A comparison of the total number of targets and the number of strongly positively correlated targets (correlation coefficients ≥ 0.6) of each regulator**.

**Regulator**	**Total number of targets**	**Strongly positively correlated targets**
ABI3	1698	47
FUS3	3076	1759
LEC1	4010	1789

*Less than half of the ABI3 and LEC1's targets are strongly positively correlated, while more FUS3 targets are strongly correlated*.

**Figure 5:**
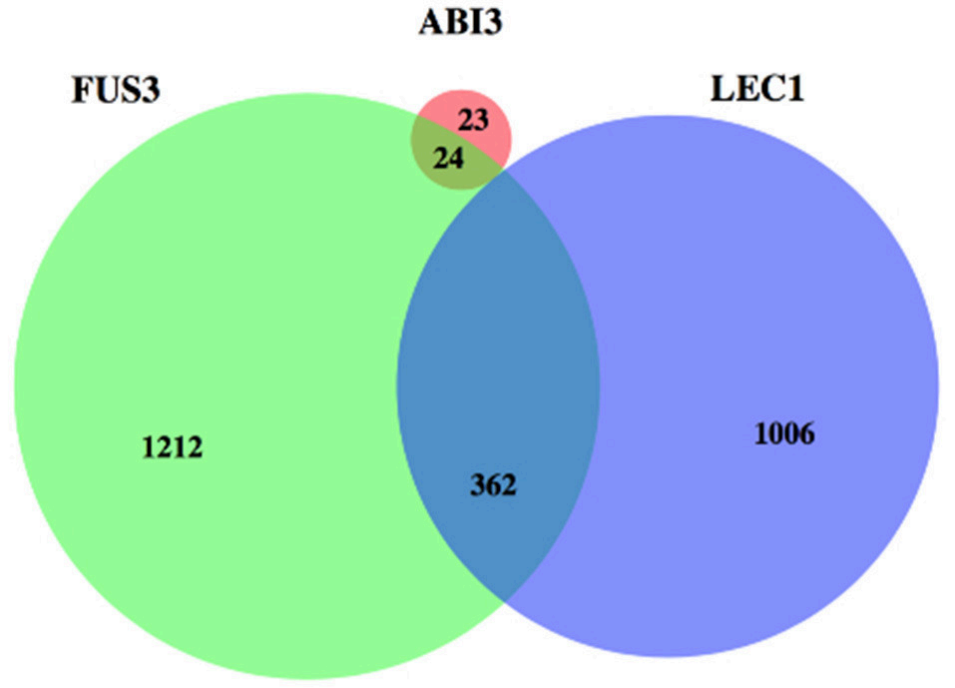
**A Venn diagram depicting the overlap between the strongly correlated targets among three regulators**. FUS3 and LEC1 have more targets than ABI3 and big overlap is shown for their targets. ABI3 has 47 targets and 24 of them are also regulated by FUS3.

The shared targets of these three regulators were identified again using the positive correlations only (Figure 5). There were 362 genes in common between targets of FUS3 and LEC1, while no overlap was found between targets of ABI3 and LEC1 under these more stringent conditions.

**Figure 6:**
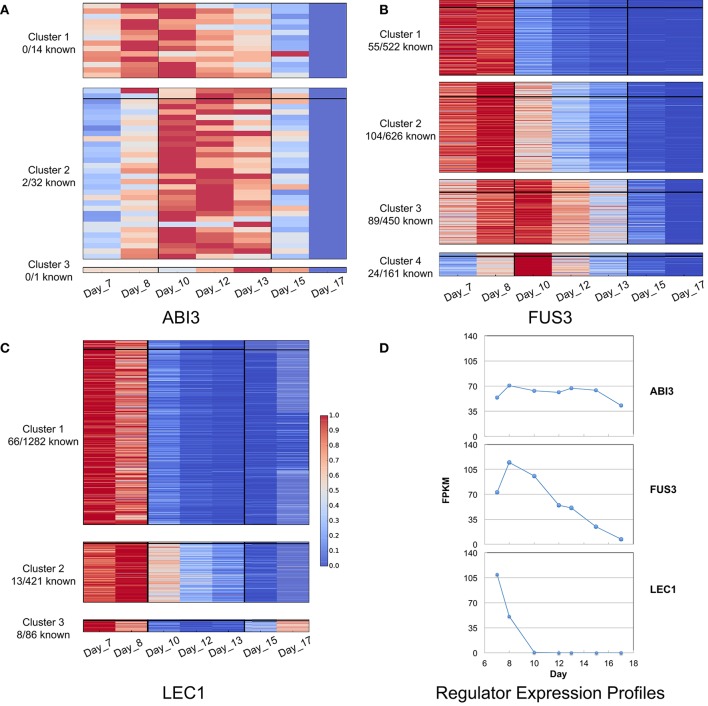
**K-means clusters of (A)** ABI3, **(B)** FUS3, and **(C)** LEC1 target genes, and the expression profiles for the three regulators **(D)**. Clusters are ordered by expression time. Three stages of seed development are involved in the gene expression: early (7 and 8 DAP), middle (10, 12, and 13 DAP), and late (15 and 17 DAP). The color scale indicates the gene expression level: red color represents high expression level, and blue color represents low expression level. A horizontal line is in each cluster, above which are the prior known targets and the remaining are predicted targets. The difference in expression profiles of the regulators may lead to different expression patterns of the target genes.

The temporal gene expression data covers three major stages in seed development: (i) early maturation (7 and 8 DAP), (ii) middle maturation (10, 12, and 13 DAP), and (iii) late maturation/early desiccation (15 and 17 DAP). Clustering all targets (including predicted and previously known targets) based on their expression profiles facilitated associating targets with specific phases of seed development. Three clusters were obtained for ABI3 and LEC1, and four clusters were obtained for FUS3 (Figure 6). All three regulators have targets that are most highly expressed at early and middle maturation stages. The only exception was LEC1 with targets in cluster 3 that showed high expression levels at the early and late maturation stages. In addition, known targets are present in each cluster, except for ABI3-assoctiated clusters 1 and 3 with no known targets.

To further evaluate the prediction results, the FIMO algorithm was used to separate all inferred targets into direct and indirect targets based on the presence of validated TF binding sites in the promoter regions. Our binding site study was limited to ABI3 and FUS3, because LEC1 is not in the CIS-BP database (Table 4). Secondary TFs were found among the direct targets in each cluster, and their binding motifs were also searched against the CIS-BP database. For example, in the FUS3-related cluster, 360 indirect targets contain the binding site in this cluster. The secondary TF AT1G01260 has a known binding motif, and, according to our inference, this gene is only controlled by FUS3.

**Table 4:** **The number of direct and indirect targets for ABI3 and FUS3, and the number of targets that overlap with GeneMANIA associations**.

**Regulator**	**Targets**	**Cluster 1**	**Cluster 2**	**Cluster 3**	**Cluster 4**
ABI3	Direct in known	0	2	0	N/A
	Indirect in known	0	0	0	N/A
	Direct in predict	2	2	18	N/A
	Indirect in predict	12	28	1	N/A
	Overlap with GeneMANIA	2	5	0	N/A
FUS3	Direct in known	9	16	15	4
	Indirect in known	46	88	74	20
	Direct in predict	40	37	36	16
	Indirect in predict	427	485	325	121
	Overlap with GeneMANIA	3	7	8	4
LEC1	Overlap with GeneMANIA	30	6	2	N/A

*The direct and indirect targets were obtained from FIMO. LEC1 does not have known binding site in the CIS-BP database, so only ABI3 and FUS3 binding sites were studied. For each regulator, the table shows the number of targets that have the binding sites in known and predicted connections, respectively*.

### Comparison of target genes predicted by the Beacon GRN inference tool with those identified in genemania for ABI3, LEC1, and FUS3

The predictions of the trained SVM model for regulator-target interactions were compared with those from GeneMania for each of the three LAFL regulators in developing Arabidopsis embryos. The Beacon GRN inference tool presented here is trained based on ChIP-Seq data. GeneMania-related gene-gene relationships are based on multiple resources (in this case, only co-expression and genetic and physical interactions were chosen), but results from ChIP-Seq data are not included in GeneMania yet. Therefore, only a partial overlap between our predictions and gene-gene relationships from GeneMania was expected.

To compare the predicted associations between our model and GeneMania-based relationships, the following steps were performed for each regulator. First, the predicted target genes showing a positive correlation (>0.6) with the selected regulator were extracted. Second, the list of these genes was compared with the list obtained for each regulator from GeneMania. This analysis, as shown in Table 4, resulted in the detection of 7 (11%), 22 (1%), and 38 (3%) genes that are positively regulated by ABI3, FUS3, and LEC1 based on both the Beacon GRN tool and GeneMania.

### Inference of genes negatively correlated with ABI3 and FUS3

The LAFL regulators ABI3, FUS3, and LEC1 are known to positively influence expression of the corresponding target genes, encoding various enzymes and regulatory proteins involved in distinct aspects of seed development and metabolism (Jia et al., 2014). However, close examination of the clustering results revealed that a substantial number of genes containing the Sph/RY regulatory motifs in their promoters (recognized by the B3 domains of ABI3 and FUS3) and confirmed binding of these LAFL regulators showed negatively correlating (*R*^2^ > 0.6) expression patterns with the patterns of these LAFL TFs (Table S1). For ABI3, 11 such genes were found in cluster 2 and 34 in cluster 3. Interestingly, the trends of genes in cluster 3 were more highly correlated with the expression pattern of *ABI3* than the trends in cluster 2 (average *R*^2^ = −0.78 ± 0.05 and −0.63 ± 0.02 for clusters 3 and 2, respectively, student's *t*-test 1.7 E^−15^). As a comparison, only 2 and 4 genes with confirmed binding of ABI3 to their promoters and trends positively correlating with ABI3 were found in clusters 1 and 2, respectively (Table S2). For FUS3, 11 and 21 genes with negatively correlating trends were present in clusters 1 and 2, respectively. In contrast, clusters 1, 2, 3, and 4 representing positive correlations between FUS3 and its target expression profiles contained a greater number of genes (49, 53, 51, and 20, respectively). Because no *cis*-element-binding information is available for LEC1, our further analyses focused only on predicted and experimentally confirmed ABI3 and FUS3 targets.

Negatively correlating trends can be explained either by (i) repression of gene expression by these LAFL regulators or (ii) combinatorial involvement of other TFs (repressors that co-express with LAFL TFs and could override the positive influence of the LAFL regulators, leading to negative correlations between expression patterns of the LAFL TFs and their target genes). In both cases, some functional connection among the targets is expected as TFs, in general, would target genes of specific functions. As such, it is not feasible to distinguish these scenarios without experimentation.

To further investigate potential functional relationships among these negatively correlated genes (*R*^2^ < −0.6), gene functions were assessed manually using TAIR 10-based functional annotations of genes within each cluster representing negative correlations (Table S1). GO enrichment analysis could not be performed due to an insufficient number of genes in individual clusters. Five (out of 11) ABI3 targets that had negatively correlated trends and were present in cluster 2 represented genes involved in transcriptional and post-transcriptional regulation. Three genes were previously uncharacterized, and 3 genes had distinct functions. The majority of 34 ABI3 targets in cluster 3 shared three basic biological functions, including (i) phytohormone signaling and transcriptional and post-transcriptional regulation (11 genes), (ii) redox regulation and energy metabolism (8 genes), and (iii) metabolism (6 genes). Seven genes had no known function, while 2 genes did not fall into any of the three functional categories. In the case of FUS3 negatively correlated targets, cluster 1 contained 4 genes involved in transcriptional and post-transcriptional regulation, while 4 genes had no known function and 3 genes had diverse functions. In FUS3-related cluster 2 (21 genes), 7 genes were related to transcriptional and post-transcriptional regulation, 4 genes to redox regulation and energy metabolism, 3 genes to cell wall metabolism, 6 genes had no known function, and 1 gene (AT5G14120) encoded a general substrate transporter. In summary, at least one functional category was identified for each cluster and only a small proportion of genes of known function had functions unrelated to the ones in the major functional categories.

We also pursued potential combinatorial involvement of other TFs that could act as repressors of FUS3 targets. There are not many known negative regulators involved in seed development (Jia et al., 2013). One of these repressors is VIVIPAROUS1/ABI3-LIKE1 (VAL1), which is known to repress genes involved in the embryonic program (Schneider et al., 2016) and is also positively regulated by FUS3 (Wang and Perry, 2013). *VAL1* was not differentially expressed above the cutoff (see Section Materials and Methods, above), so the *VAL1* gene was absent from any clusters. *VAL1* has four functional domains responsible for epigenetic and transcriptional regulatory functions of this protein, one of which is a B3 domain that recognizes the Sph/RY motif (Jia et al., 2013), that could interfere with FUS3-mediated transcriptional activation and be responsible for negatively correlated trends of some of the predicted and known FUS3 targets. To test this possibility, the list of genes from FUS3 clusters 1 and 2 was compared to the list of predicted VAL1 targets (Schneider et al., 2016). Only 2 genes that were negatively correlated with FUS3 (AT1G01190 and AT1G01580 encoding a cytochrome P450 monooxygenase CYP78A8 and ferric reduction oxidase FRD1, respectively) were identified, which could be attributed to the weak correlation between *VAL1* and FUS3 expression patterns.

## Discussion

We have developed the Beacon GRN inference tool, a supervised machine learning method based on a local SVM approach, to infer complex GRNs representing gene-regulator interactions occurring in developing Arabidopsis embryos from gene expression data and known regulatory relationships used as a prior knowledge. The local SVM approach with RBF kernel was chosen based on a performance comparison with the global SVM approach and the unsupervised method CLR. CLR does not take into account any known interactions and performs worse than supervised methods. The global SVM approach makes an assumption that all TFs regulate their downstream targets in the same way, and it performs worse than the local SVM models. A linear SVM kernel generates a linear hyperplane to separate positive and negative examples, which is less flexible than the non-linear kernel RBF. We concluded that the local SVM approach with RBF is the most suitable method to infer GRNs related to embryo development. The resulting Beacon GRN inference tool decomposes the problem of inferring a network into three different subproblems with the goal of identifying targets of each of the three regulators.

The Beacon GRN inference tool enabled the prediction of targets controlled by one or more regulators. There were 521 genes predicted to be regulated by all three genes, but a number of shared targets were found between any two of the regulators. Although, the actual gene-regulator relationship predictions remain to be experimentally validated, they provide a useful resource for plant biologists. An unexpected finding was the identification of potential negatively regulated targets of ABI3 and FUS3 that shared functions in signaling and gene expression and redox regulation. he findings reported here were compared with a recently published RNA-Seq data set documenting gene expression in Arabidopsis seeds during the final stages of development (days 15, 17, 21; Gonzalez-Morales et al., 2016). The comparison revealed that 45 transcripts that showed a negative correlation with the expression of ABI3 also showed higher expression in an abi3 mutant compared to the wild type at least one of the time points studied in that report, providing biological validation of the computational approach adopted here. Four of these 45 transcripts have a binding site for ABI3. Although it was not possible to distinguish direct repression of gene expression by these LAFL regulators from potential combinatorial involvement of secondary TFs acting as repressors, these two scenarios can be tested experimentally on specific gene-regulator interaction predictions. Moreover, our method of TF target prediction can be easily expanded to infer regulatory networks for other biological processes in different plants by replacing the data source.

As with many inference models, there is a limitation based on the initial data set used to make predictions. The prediction accuracy of the Beacon GRN inference tool could be improved by adding known TF-target pairs as such information becomes available. In addition, the AUC was computed by assuming that the known interactions are accurate and do not include undiscovered relationships. One of the limitations of our Beacon GRN inference tool is its inability to predict regulatory relationships with no prior known relations. The performance of the Beacon tool is dependent upon a list of known target genes, and, as such, an incomplete list will produce poor GRN prediction results. A possible future direction to address this challenge is to implement semi-supervised approaches yielding hybrid models based on prior knowledge when available but also able to accommodate parts of data with missing knowledge.

## Author contributions

Conceived and designed the experiments: YN, SL, RG, LH. Performed the experiments: YN, DA, HE. Analyzed the data and wrote the paper: YN, DA, HE, EC, SL, RG, LH.

## Funding

This work was supported by NSF grant DBI-1062472 and the Genomics, Bioinformatics, and Computational Biology doctoral program at Virginia Tech. Funding for this work was also provided by the Virginia Agricultural Experiment Station and the Hatch Program of the NIFA, USDA.

### Conflict of interest statement

The authors declare that the research was conducted in the absence of any commercial or financial relationships that could be construed as a potential conflict of interest. The reviewer MM declared a past collaboration with one of the authors SL to the handling Editor, who ensured that the process met the standards of a fair and objective review.
