# Supplementary figures and images for: A Machine Learning Approach to Predict Gene Regulatory Networks in Seed Development in Arabidopsis
Source: Front Plant Sci. 2016 Dec 23;7:1936. doi: 10.3389/fpls.2016.01936 (PMC5179539; doi:10.3389/fpls.2016.01936)

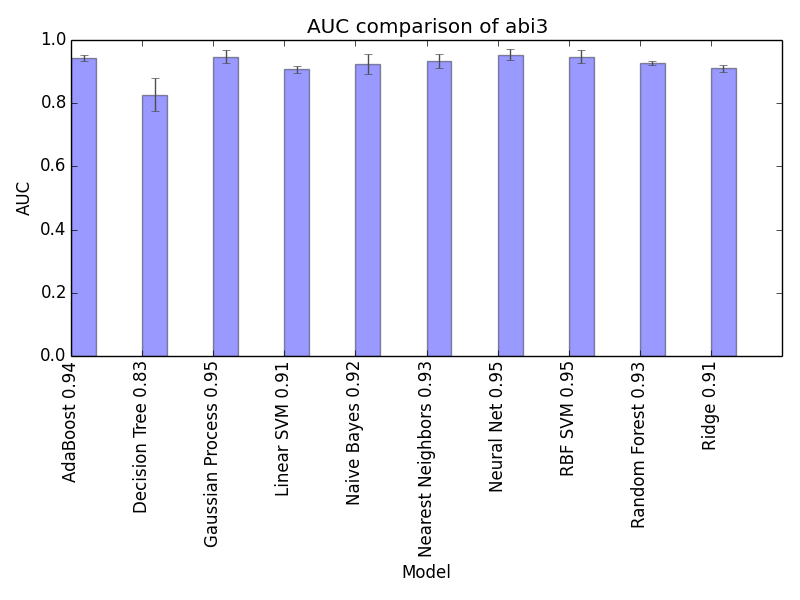

Supplement: Supplementary file 3 [file Image1.PNG]

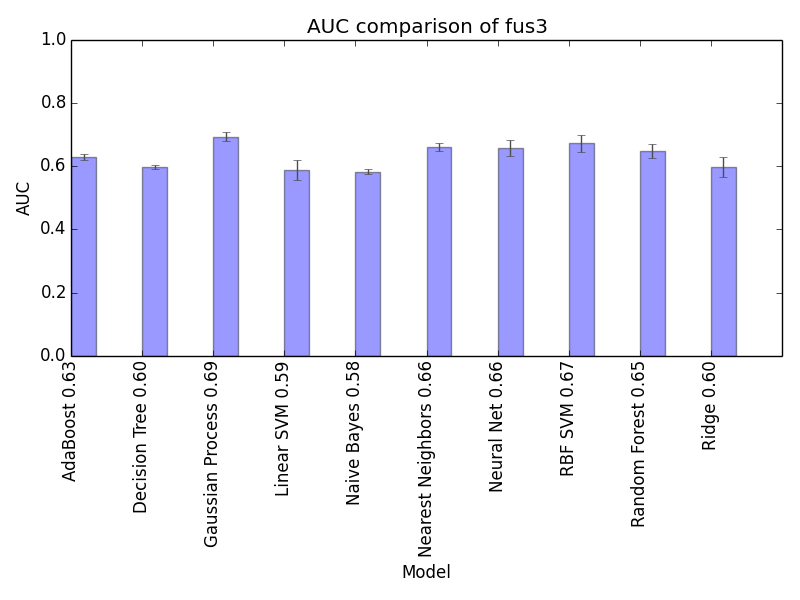

Supplement: Supplementary file 4 [file Image2.PNG]

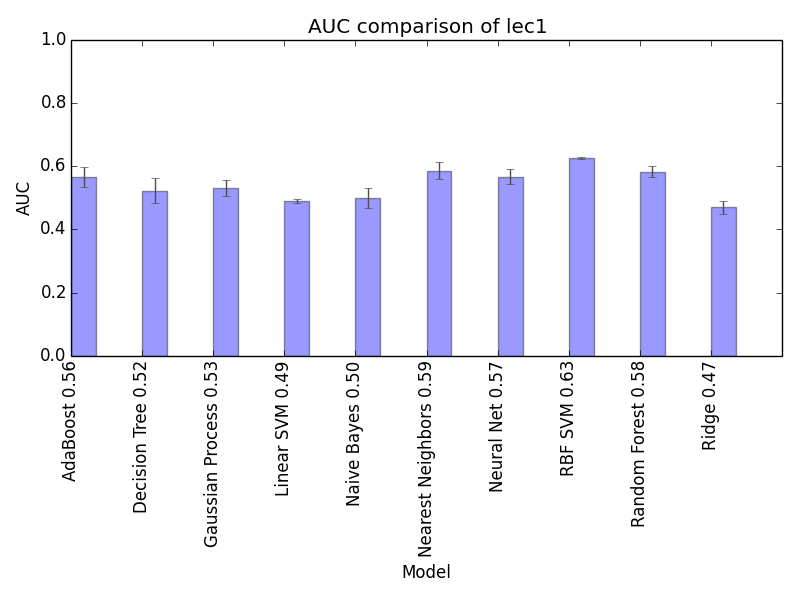

Supplement: Supplementary file 5 [file Image3.PNG]

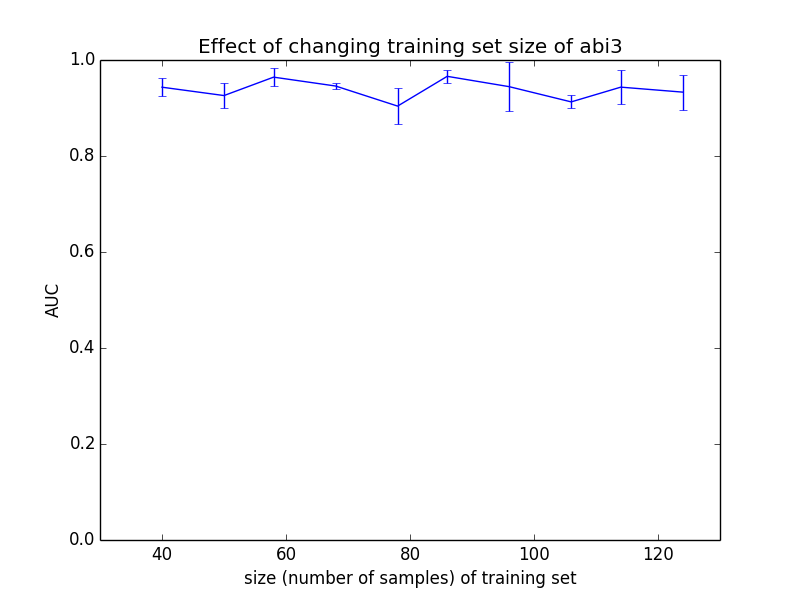

Supplement: Supplementary file 6 [file Image4.PNG]

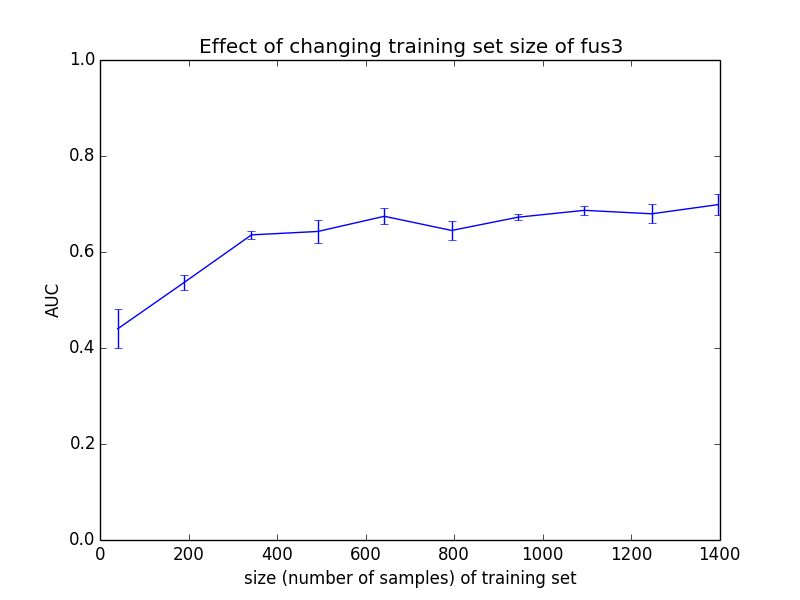

Supplement: Supplementary file 7 [file Image5.PNG]

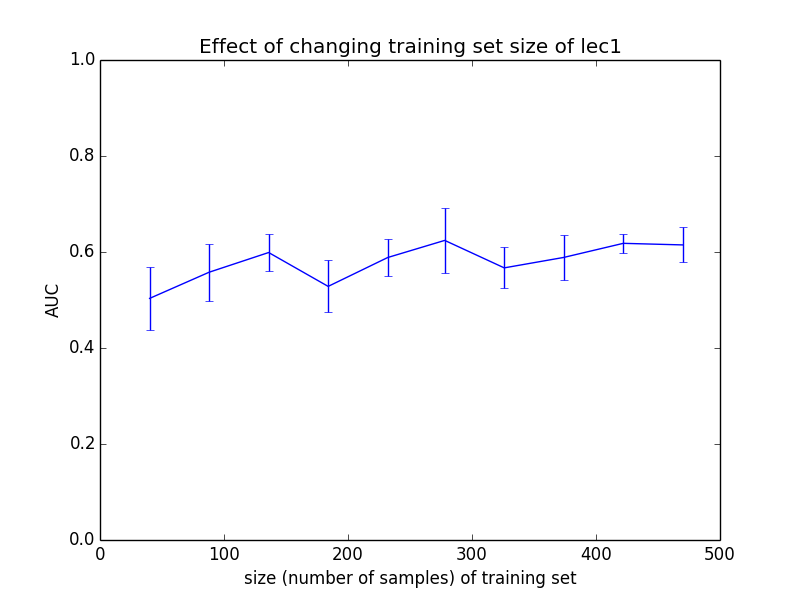

Supplement: Supplementary file 8 [file Image6.PNG]
